# Mechanical transmission of impulsive instrument-assisted spinal manipulation: contact-site-dependent biomechanical coupling in the *in vivo* human lumbar spine

**DOI:** 10.3389/fbioe.2026.1849593

**Published:** 2026-08-13

**Authors:** Christopher J. Colloca, Robert Gunzburg, Mostafa Afifi Hegazy, Marek Szpalski

**Affiliations:** 1 Department of Kinesiology, College of Health Solutions, Arizona State University, Phoenix, AZ, United States; 2 International Spine Research Foundation, Dexterville, NY, United States; 3 Cavell Spine Centre, Edith Cavell Hospital, Brussels, Belgium; 4 Department of Kinesiology, Southwest Minnesota State University, Marshall, MN, United States; 5 Department of Orthopaedics, Hospital Moliere Longchamp, Brussels, Belgium

**Keywords:** accelerance, accelerometry, biomechanics, contact site, electromyography, instrument-assisted, lumbar spine, spinal manipulation

## Abstract

**Background:**

Spinal manipulation is widely used in clinical practice, yet how contact site selection influences mechanical transmission through spinal structures remains poorly understood. This exploratory study characterizes the biomechanical transmission of impulsive spinal manipulative thrust (SMT) forces through the *in vivo* human lumbar spine, examining how contact site and vertebral level alter the mechanical and neuromuscular response.

**Methods:**

Four patients undergoing elective lumbar surgery were instrumented with triaxial accelerometers on intraosseous Steinmann pins at L2 and L4, and bilateral needle electromyographic (nEMG) electrodes. An Impulse iQ adjusting instrument delivered impulsive SMTs to six contact points in randomized order: L3 spinous process, L5 spinous process, and bilateral L2 and L4 facet joints totaling 1,549 usable for force-dependent analyses after excluding one patient with an instrument force malfunction. Patient-level aggregation (n = 3–4) served as the primary statistical approach.

**Results:**

Two patterns of mechanical transmission were observed. Spinous process contacts produced larger posterior-anterior vertebral acceleration at L2 in all patients (14.95 ± 10.97 vs. 6.75 ± 4.62 g, patient-level *P* = 0.066), while facet contacts produced larger cranial-caudal acceleration at L4 (2.50 ± 2.27 vs. 0.94 ± 0.59 g). L3 spinous contacts produced substantially greater L2 posteroanterior acceleration than L5 contacts (21.3 ± 10.9 vs. 8.2 ± 5.8 g, patient-level *P* = 0.021), with proximity-dependent inter-vertebral attenuation reflected by transfer ratios of 0.094 (L3) versus 0.619 (L5). Facet contacts showed no significant bilateral asymmetry at the patient level.

**Conclusion:**

These exploratory findings suggest that contact site selection influences the transmission pathway architecture and directional character of the vertebral mechanical response during impulsive SMTs. These biomechanical observations may help generate hypotheses for future clinical studies examining the relationship between contact site selection and therapeutic outcomes.

## 1 Introduction

Instrument-assisted spinal manipulation has become one of the most widely used techniques for delivering spinal manipulative therapy (SMT) in chiropractic and manual medicine practice (Colloca et al., 2006; Kawchuk and Herzog, 1993; Pickar et al., 2007). Handheld mechanical adjusting instruments, such as the Impulse iQ (Neuromechanical Innovations, Phoenix, Arizona, USA), deliver precisely controlled impulsive forces with thrust durations on the order of 2 milliseconds and peak forces ranging from approximately 100–400 N (Colloca et al., 2006; Colloca et al., 2008). These devices enable high reproducibility and force metering that are difficult to achieve with manual thrust delivery (Colloca et al., 2006; Descarreaux et al., 2005).

Despite decades of clinical and biomechanical investigation, fundamental questions remain regarding how mechanical forces applied to the spine during SMT are transmitted through vertebral and perivertebral structures to produce their biomechanical and physiological effects (Pickar et al., 2007; Colloca et al., 2000; Keller et al., 2003a; Pickar, 2002). Early *in vivo* studies demonstrated that impulsive SMT produces measurable acceleration at vertebrae adjacent to the contact site, with responses varying by force amplitude and vertebral level (Colloca et al., 2000; Keller et al., 2003a). Pickar (Pickar, 2002) outlined the neurophysiological mechanisms by which mechanical input during SMT may modulate paraspinal muscle spindle afferent discharge, providing a theoretical framework linking mechanical transmission to neuromuscular responses.

In clinical practice, clinicians select the vertebral contact site based on the perceived type of segmental dysfunction. Spinous process (SP) contacts are traditionally chosen when the primary dysfunction is sagittal (flexion-extension), while facet joint contacts are selected for coupled rotational or lateral flexion restrictions (Colloca et al., 2006; Colloca et al., 2000). However, this decision is largely guided by clinical convention from global range of motion and palpatory assessment rather than quantitative biomechanical evidence regarding how the contact site modulates force transmission through the spinal complex.

The anatomical architecture of the two contact sites differs substantially. The spinous process is a midline structure coupled to adjacent vertebrae through the supraspinous and interspinous ligament complex, which transmits forces along the posterior sagittal plane. The facet (zygapophyseal) joint, by contrast, is a laterally positioned synovial articulation situated deep to the paraspinal musculature, with an orientation that varies from near-sagittal at upper lumbar levels to more coronal at lower levels. These structural differences suggest that the two contact sites may couple mechanical energy into the vertebral column through different pathways, potentially producing different acceleration response profiles at adjacent segments. Cadaveric studies by Funabashi and colleagues (Funabashi et al., 2018; Funabashi et al., 2016; Funabashi et al., 2022) have demonstrated that the site of SMT application alters the loading distribution across spinal tissues, with different contact points producing distinct patterns of facet joint, disc, and ligament loading.

Previous *in vivo* studies of impulsive SMT have focused primarily on isolated measurements of peak force, vertebral acceleration, or spinal stiffness at individual contact sites (Colloca et al., 2000; Keller et al., 2003a; Keller et al., 2003b; Colloca et al., 2005; Colloca and Keller, 2001). The driving-point posterior-anterior (PA) stiffness index, computed as stylus accelerance (the ratio of stylus acceleration to applied force), has been reported at 5.5–7.0 kg^-1^ for lumbar spinous process contacts in intact subjects (Colloca and Keller, 2001). Cadaveric studies using robotic serial dissection protocols have further characterized the internal tissue loading during SMT (Kawchuk et al., 2010), while animal models have demonstrated that paraspinal muscle spindle responses are graded by distance from the contact site (Pickar et al., 2007; Reed and Pickar, 2015). Triano and colleagues (Triano et al., 2012) have characterized the force-time profiles of manual SMT delivery, and Descarreaux and colleagues (Descarreaux and Dugas, 2010) have examined how force parameters influence spinal stiffness measurements, providing important context for understanding the mechanical input during both manual and instrument-assisted techniques.

Despite this body of work, no study has characterized the complete mechanical transmission chain from the contact instrument through the vertebral column *in vivo*, nor has any study systematically examined how the choice of contact site modulates this transmission. Understanding the biomechanical coupling between the point of force application and the vertebral response is a necessary precursor to evaluating whether different contact site selections produce clinically meaningful differences in therapeutic outcome.

The purpose of this exploratory, proof-of-concept study was to characterize the mechanical transmission pathways of impulsive spinal manipulation as a function of contact site and vertebral level in the *in vivo* human lumbar spine. Specifically, we aimed to: (1) quantify the driving-point accelerance (stylus acceleration/force ratio) at spinous process and facet joint contacts; (2) characterize the triaxial acceleration response at adjacent vertebrae; (3) compute inter-vertebral transfer ratios to quantify the extent of mechanical transmission between instrumented segments; and (4) quantify the associated neuromuscular responses via needle electromyography. Given the small sample inherent in this intraoperative protocol, all analyses are presented as exploratory and hypothesis-generating.

## 2 Materials and methods

### 2.1 Participants

Four patients (3 female, 1 male; age range 54–70 years, mean 61.5 ± 5.7 years) undergoing elective posterior lumbar decompressive surgery at Eeuwfeestkliniek Hospital (Antwerpen, Belgium) were enrolled. All patients provided written informed consent prior to participation. The study protocol was approved by the institutional ethics committee of Eeuwfeestkliniek Hospital. General anesthesia was induced with propofol and maintained with sevoflurane and remifentanil infusion, with neuromuscular blockade allowed to dissipate prior to data collection to preserve reflexive EMG responses. Patients were positioned prone on a standard surgical frame in a lordotic posture consistent with the operative position.

### 2.2 Instrumentation

**FIGURE 1:**
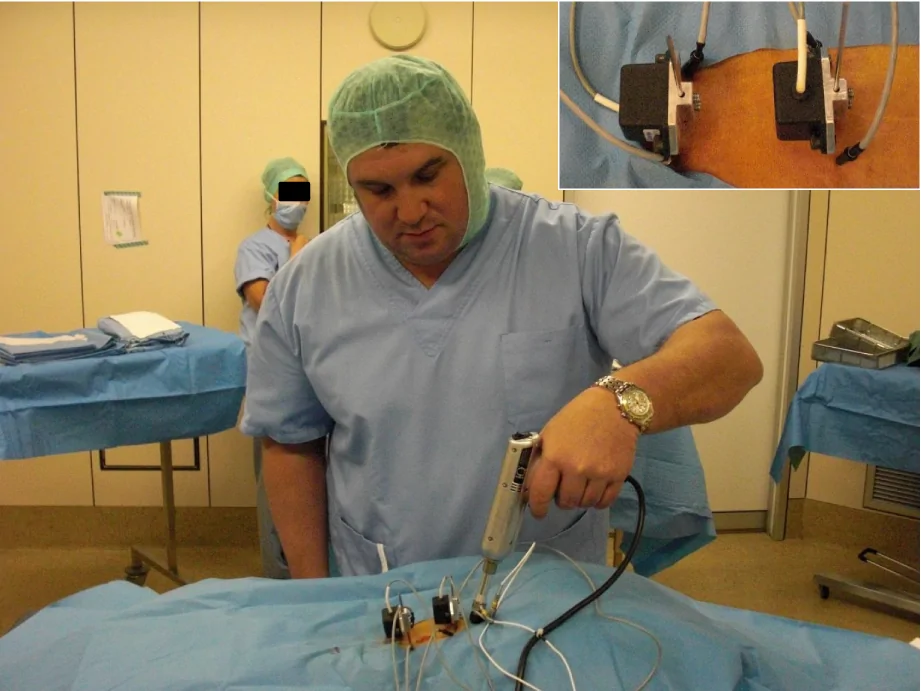
Intraoperative view of the experimental setup during an L5 SMT trial. Triaxial accelerometers were mounted on intraosseous pins at the L2 and L4 spinous processes and needle EMG electrodes were placed in the paraspinal musculature at L2 and L4, bilaterally. The Impulse iQ adjusting instrument® (Neuromechanical Innovations, Phoenix, Arizona, USA) was applied to the exposed lumbar spine at L3 or L5 contact sites (spinous process, left facet, or right facet). Figure contains images of the author(s) only.

Prior to surgical exposure of the lumbar spine, triaxial accelerometers (Model CXL10HF3, Crossbow Technology, San Jose, CA; bandwidth 0.3–10,000 Hz, range ±10 g) were rigidly mounted on intraosseous Steinmann pins inserted into the spinous processes of L2 and L4 identified by fluoroscopy (Figure 1). The accelerometer axes were oriented as follows: x-axis = medial-lateral (ML), y-axis = posterior-anterior (PA), and z-axis = cranial-caudal (CC). Bipolar needle electromyographic (nEMG) electrodes were inserted bilaterally into the lumbar paraspinal musculature at the L2 and L4 levels, yielding four EMG channels designated as superior left (L2 left), superior right (L2 right), inferior left (L4 left), and inferior right (L4 right).

An Impulse iQ adjusting instrument® (Neuromechanical Innovations, Phoenix, Arizona, USA) served as the medical device for spinal manipulation force delivery (Figure 1). The instrument was equipped with a force transducer (Model 201A03, PCB Piezotronics, Depew, NY) mounted at the stylus tip together with and a uniaxial accelerometer (Model 305A04, PCB Piezotronics) mounted in series. All 12 analog channels (1 force, 1 stylus accelerometer, 6 vertebral accelerometer axes, 4 nEMG) were acquired simultaneously using a Biopac MP150 data acquisition system (Biopac Systems, Inc., Goleta, CA) at sampling rates of 100 kHz for accelerometer and force channels and 5 kHz for EMG channels.

### 2.3 Experimental protocol

A repeated-measures randomized factorial design was employed with two vertebral levels (L3, L5) crossed with three contact types (spinous process, left facet, right facet) and three force settings (Low ≈ 96 N, Medium ≈ 266 N, High ≈ 328 N). Each experimental condition consisted of a pulse train of consecutive impulses delivered in randomized trial order across the six contact sites. Recording files were separated by at least 1 min to allow tissue recovery between conditions.

For spinous process contacts, the instrument stylus tip (3.2 mm diameter, spherical rubber contact surface) was positioned over the midline dorsal prominence of the spinous process at L3 or L5, localized as the immediate midline bony prominence inferior to the radiographically identified L2 and L4 levels for bone pin implantation. For facet contacts, the stylus was positioned 2.5 cm lateral to the spinous process contact on the skin overlying the inferior articular process of the facet joint of the target level, localized by direct visualization and palpation. All thrusts were directed in the posteroanterior (PA) and direction perpendicular to the plane of the operating table with a 60° cranial angle consistent with standard clinical application of the Impulse iQ® instrument. The instrument handle was maintained in a consistent orientation by the same operator (CJC) throughout all data collection to minimize variability in application angle across conditions.

In total, 3,234 thrust samples were obtained across 69 valid recording files from the four patients. Of these, 1,549 thrusts from Patients 02–04 were usable for force-dependent analyses (see Section 2.4 for exclusion criteria). The distribution of force-usable thrusts by contact type was: spinous process, 535 (L3: 275, L5: 260); facet joint, 1,014 (L2 left: 272, L2 right: 225, L4 left: 255, L4 right: 262).

### 2.4 Data quality and exclusions

Signal processing calibration utilized voltage offset and scale parameters from the calibration block embedded in each data acquisition file. Patient 01’s force transducer produced anomalous values (approximately 5–9 N across all force settings, compared with expected values of 100–400 N), indicating an instrument force transducer malfunction during that patient’s data collection. Patient 01 was therefore excluded from all force-dependent analyses (accelerance, vertebral accelerance), yielding 1,549 force-usable thrusts from Patients 02–04. Patient 01 data were retained for acceleration-only analyses where force was not required as a denominator.

EMG channel availability varied across patients and conditions. Only the L4 right (inferior right) channel provided complete data across all 1,549 force-usable thrusts (100% availability). The remaining three EMG channels (L2 left, L2 right, L4 left) were usable in 728 of the 1,549 force-usable thrusts (47%), with data limited to Patients 03 and 04.

The triaxial accelerometers (Crossbow CXL10HF3, nominal range ±10 g) produced readable data across all conditions; however, acceleration responses occasionally exceeded the nominal measurement range. For L2, 14.2% of thrusts exceeded 10 g peak-to-peak on at least one axis (predominantly the posteroanterior axis under spinous process contacts at the highest force setting). For L4, fewer than 2% of thrusts approached the measurement ceiling. A sensitivity analysis excluding these ceiling-adjacent thrusts did not change the direction or significance of any patient-level comparison, as the affected thrusts were distributed proportionally across contact types within each patient. The affected thrusts were concentrated in the posteroanterior axis under spinous process contacts at the two highest force settings (Settings 3 and 4) and were distributed proportionally across all three patients. Because saturation predominantly affected L2 PA acceleration under spinous contacts, the effect is to underestimate the true L2 PA response for spinous contacts, meaning that the reported facet-versus-spinous differences at L2 PA are conservative. The sensitivity analysis confirmed that the largest change in any patient-level Cohen’s d after exclusion of ceiling-adjacent thrusts was less than 0.1, and all qualitative conclusions remained identical.

### 2.5 Data processing

Peak-to-peak (P2P) amplitudes were computed for all channels over a 250-millisecond window bracketing each thrust onset. Thrust onset was detected by threshold-crossing on the force channel, defined as the first sample exceeding three times the baseline standard deviation computed from a quiescent pre-thrust interval. The detection algorithm was validated against manual identification in the manufacturer’s software (AcqKnowledge). No additional digital filtering was applied beyond the hardware anti-aliasing filters inherent in the data acquisition system, preserving the full bandwidth of the impulsive response.

### 2.6 Derived transmission metrics

Four derived metrics were computed to characterize mechanical transmission:

1. Stylus accelerance was defined as the ratio of P2P stylus acceleration (m/s^2^) to P2P applied force (N), expressed in kg^-1^. Accelerance was computed as a time-domain P2P ratio, providing a time-domain approximation of the frequency-dependent accelerance transfer function. This metric characterizes the driving-point mechanical impedance properties of the contact site.
2. Inter-vertebral transfer ratio was defined as the ratio of L4 z-axis P2P acceleration to L2 z-axis P2P acceleration (L4_z_P2P/L2_z_P2P). Values less than 1.0 indicate attenuation of cranial-caudal acceleration from the superior to inferior instrumented vertebra.
3. Axial dominance index was computed for each accelerometer axis as the ratio of that axis P2P amplitude to the sum of all three axes (e.g., z-axis dominance = Z_P2P/[Z_P2P + Y_P2P + X_P2P]). This metric quantifies the directional distribution of the acceleration response.
4. Vertebral accelerance was defined as the ratio of vertebral acceleration (m/s^2^) to P2P applied force (N), expressed in kg^-1^. This metric extends the accelerance concept to the vertebral response, providing a measure of the force-normalized mechanical transmission to the instrumented vertebrae.

To clarify the biomechanical metrics used throughout: (1) Stylus accelerance is the acceleration measured at the instrument tip per unit applied force, reflecting the driving-point mechanical impedance of the tissue directly beneath the contact; (2) Vertebral accelerance is the acceleration measured at a remote vertebral pin per unit applied force, reflecting the transfer impedance through intervening spinal structures; (3) The inter-vertebral transfer ratio is the ratio of L4 to L2 acceleration (dimensionless), characterizing how much of the L2 response is transmitted across the intervening motion segments to L4.

### 2.7 Statistical analysis

Patient-level aggregation was adopted as the primary analytical approach. For each comparison, per-patient means were computed for each experimental condition, and paired tests (paired *t*-tests) were performed on these patient-level summary statistics. This approach treats the patient as the experimental unit, eliminating the pseudoreplication that would arise from treating individual thrusts as independent observations. With only 3–4 patients, random-effects variance components for mixed-effects models would be unstable and difficult to interpret, and convergence may be unreliable; patient-level aggregation is therefore more transparent and conservative for small-cluster designs (Galbraith et al., 2010). Given the small number of independent experimental units (n = 3–4), all *P* values reported herein should be interpreted as descriptive summaries of the observed data rather than as confirmatory tests of biomechanical hypotheses.

Intraclass correlation coefficients (ICC) were computed using a two-way random-effects model for absolute agreement [ICC(2,1)], reflecting both inter-patient and inter-condition sources of variance. 95% confidence intervals were computed using the F-distribution method. ICC values informed the decision to aggregate at the patient level: high within-patient ICC (> 0.75) confirmed that patient-level means were reliable summary statistics, while high between-patient ICC indicated that patient identity was a meaningful grouping factor requiring aggregated analysis.

Cohen’s *d* effect sizes were computed at the patient level where feasible. No formal multiple testing correction was applied (see Section 4.1 for discussion). With so few degrees of freedom, correction methods would render essentially all comparisons non-significant regardless of effect magnitude. We therefore emphasize effect sizes and directional consistency across patients rather than relying solely on *P* values. Statistical significance was assessed at α = 0.05, two-tailed.

## 3 Results

### 3.1 Driving-point properties and stylus accelerance

**FIGURE 2:**
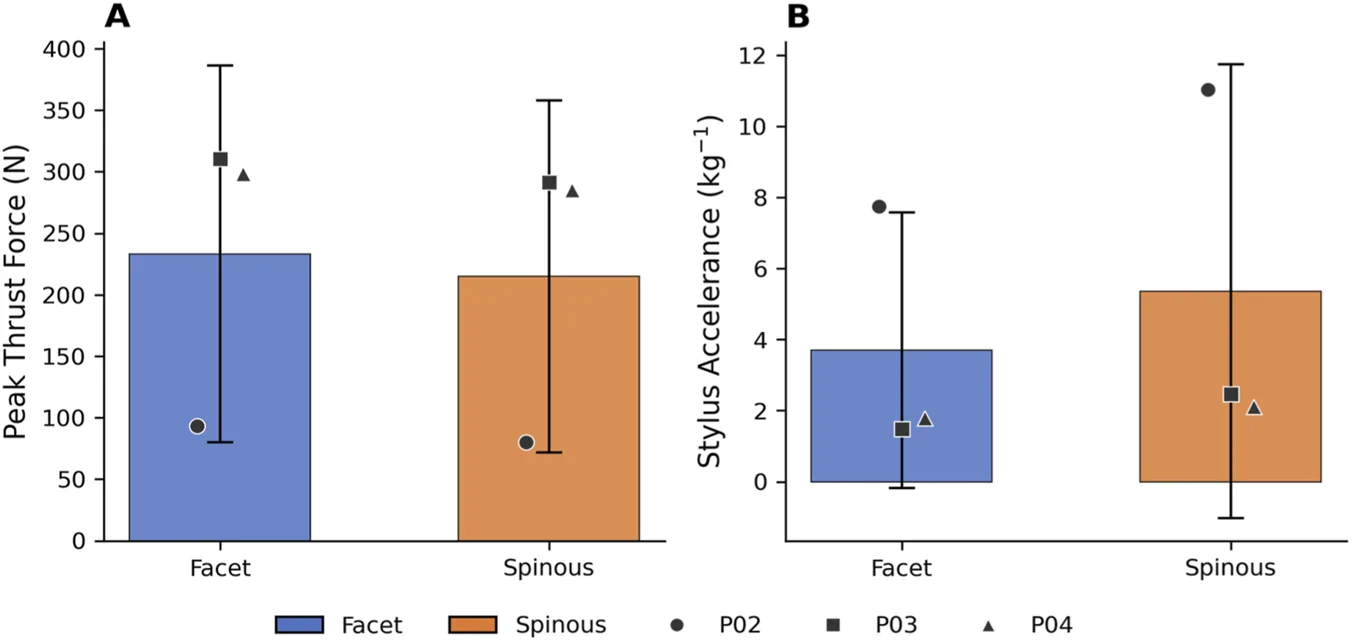
Driving-point properties by contact type (Patients 02–04, n = 1,549 thrusts). **(A)** Peak thrust force was comparable between facet (233 ± 153 N) and spinous (215 ± 143 N) contacts. **(B)** Stylus accelerance was numerically lower for facet (3.70 ± 3.87 kg^-1^) than spinous (5.36 ± 6.37 kg^-1^) contacts, consistent with published values of 5.5–7.0 kg^-1^ for the lumbar spinous process. Error bars represent ±1 SD.

Applied force differed statistically between facet and spinous contacts at the patient level (233 ± 153 vs. 215 ± 143 N, patient-level *P* = 0.016; Table 1; Figure 2), although the absolute difference was small (thrust-level Cohen’s *d* = 0.12, corresponding to an approximately 7% relative difference). The consistency of the small force difference across all three patients (each showing approximately 13–19 N higher force for facet contacts) accounts for the statistical significance despite the small practical magnitude. All subsequent accelerance and vertebral accelerance metrics are force-normalized, thereby controlling for this minor difference in applied force.

**TABLE 1:** Driving-point properties by contact type (Patients 02–04, n = 1,549 thrusts).

Variable	Facet (n = 1,014)	Spinous (n = 535)	Patient d	Patient P
Force P2P (N)	233 ± 153	215 ± 143	4.58	0.016
Stylus accelerance (kg^-1^)	3.70 ± 3.87	5.36 ± 6.37	−0.99	0.229

Values are thrust-level means ± SD. P values from patient-level paired t-test (n = 3 patients). The large patient-level d for force (4.58) reflects high inter-patient consistency (all three patients showed the same directional difference) rather than clinically meaningful magnitude; the absolute difference was approximately 15 N (∼7% of mean force).

Stylus accelerance was 3.70 ± 3.87 kg^-1^ for facet contacts and 5.36 ± 6.37 kg^-1^ for spinous process contacts. These values are consistent with previously published PA stiffness indices of 5.5–7.0 kg^-1^ reported for lumbar spinous process contacts in intact subjects (Colloca and Keller, 2001).

### 3.2 Triaxial vertebral acceleration responses

**FIGURE 3:**
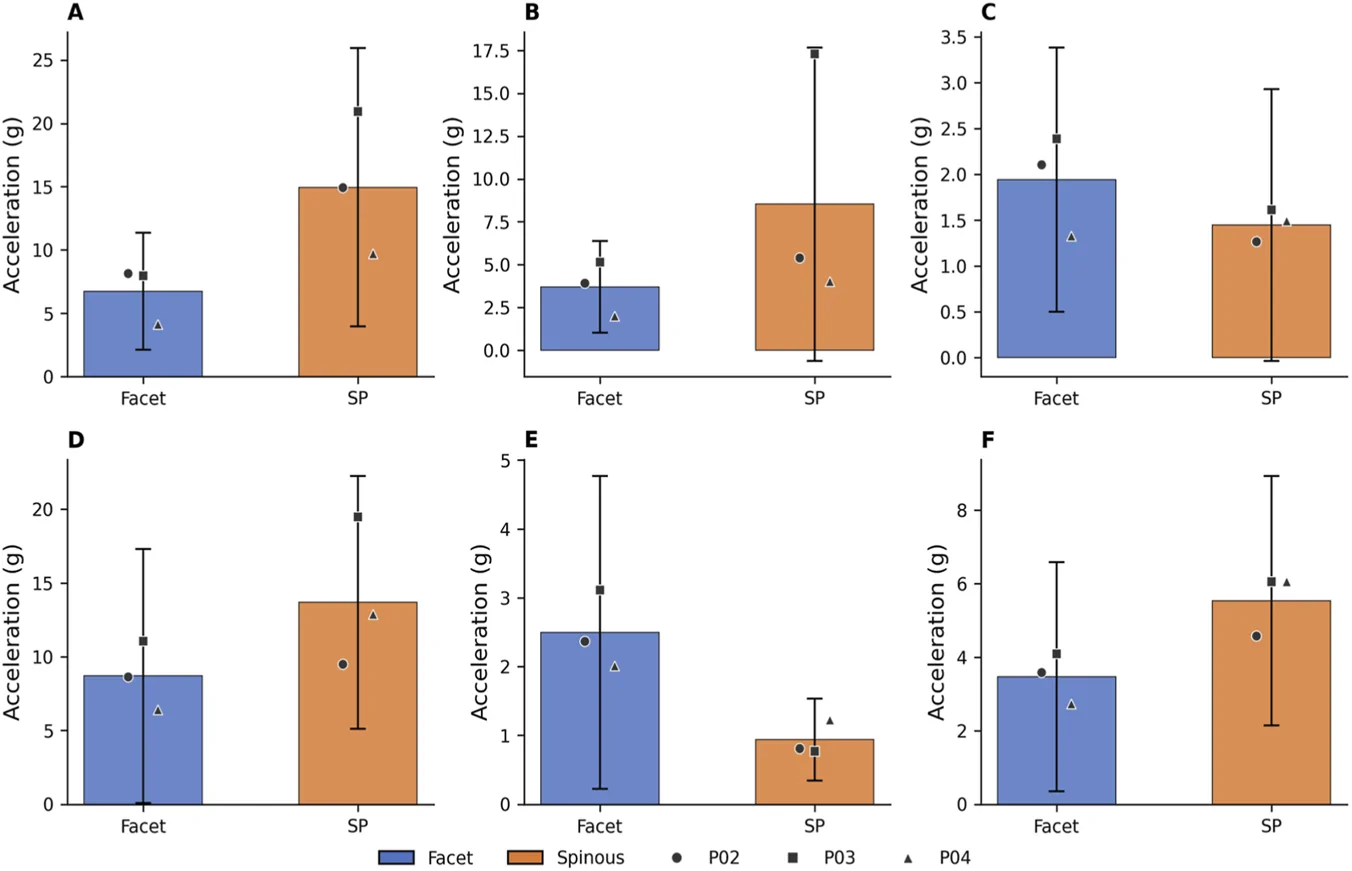
Triaxial vertebral acceleration by contact type (Patients 02–04). Upper panels show L2 (superior) pin responses; lower panels show L4 (inferior) pin responses. Axes: z = cranial-caudal (CC), y = posterior-anterior (PA), x = medial-lateral (ML). Spinous process contacts produced larger L2 PA acceleration (14.95 vs. 6.75 g), while facet contacts produced larger L4 CC acceleration (2.50 vs. 0.94 g). Error bars represent ±1 SD. Values above bars are means. **(A)** L2 PA. **(B)** L2 CC. **(C)** L2 ML. **(D)** L4 PA. **(E)** L4 CC. **(F)** L4 ML.

Spinous process contacts produced larger posterior-anterior (PA) acceleration at L2 than facet contacts in all three patients with usable force data (14.95 ± 10.97 vs. 6.75 ± 4.62 g, *d* = −2.13, patient-level *P* = 0.066; Table 2; Figure 3). Spinous process contacts also produced larger cranial-caudal (CC) acceleration at L2 (8.53 ± 9.13 vs. 3.70 ± 2.68 g, *d* = −0.87, patient-level *P* = 0.272), although the patient-level comparison did not reach statistical significance.

**TABLE 2:** Triaxial vertebral acceleration and vertebral accelerance by contact type (Patients 02–04, n = 1,549 force-usable thrusts).

Variable	Facet	Spinous	Patient d	Patient P
L2 y-axis, PA (g)	6.75 ± 4.63	14.95 ± 10.98	−2.13	0.066
L2 z-axis, CC (g)	3.70 ± 2.68	8.53 ± 9.14	−0.87	0.272
L2 x-axis, ML (g)	1.94 ± 1.44	1.45 ± 1.49	0.86	0.276
L4 y-axis, PA (g)	8.69 ± 8.61	13.69 ± 8.57	−1.34	0.146
L4 z-axis, CC (g)	2.50 ± 2.27	0.94 ± 0.59	2.01	0.074
L4 x-axis, ML (g)	3.47 ± 3.12	5.54 ± 3.39	−1.79	0.090

Values are thrust-level means ± SD. PA, posterior-anterior; CC, cranial-caudal; ML, medial-lateral. P values from patient-level paired t-test (n = 3 patients). Effect sizes computed at the patient level.

At L4, the pattern was axis-dependent. Facet contacts produced larger cranial-caudal acceleration (2.50 ± 2.27 vs. 0.94 ± 0.59 g, *d* = 2.01, patient-level *P* = 0.074), but spinous contacts produced larger posteroanterior acceleration (13.69 ± 5.07 vs. 8.69 ± 2.31 g, *d* = −1.34, *P* = 0.146) and larger medial-lateral acceleration (5.56 ± 0.85 vs. 3.47 ± 0.69 g, *d* = −1.79, *P* = 0.090). This axis-dependent pattern suggests that the two contact types channel mechanical energy along different spatial trajectories: spinous process contacts produce predominantly posteroanterior acceleration at both L2 and L4, while facet contacts produce a relatively greater cranial-caudal component at L4.

Intraclass correlation coefficients indicated moderate within-patient clustering for force (ICC = 0.544) and L2 cranial-caudal acceleration (ICC = 0.251), but minimal clustering for L4 cranial-caudal acceleration (ICC = 0.025), suggesting that L4 responses were primarily driven by within-patient variability across conditions rather than between-patient differences.

### 3.3 Multi-axial response profiles

**FIGURE 4:**
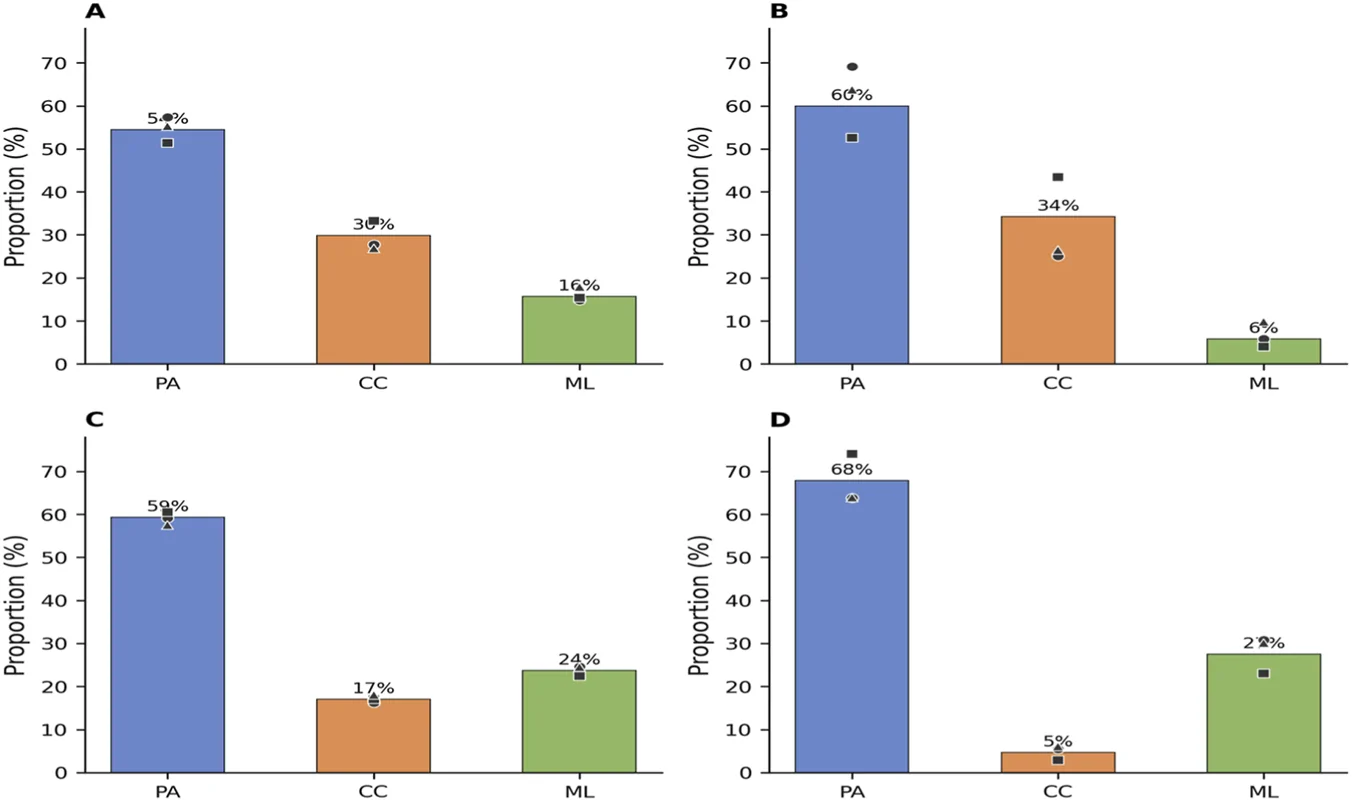
Multi-axial response profiles showing the relative contribution of each acceleration axis to total vertebral acceleration. The PA (y-axis) component dominated at both L2 and L4 (54%–68%), while the CC (z-axis) contribution was markedly larger at L2 (30%–34%) than L4 (5%–17%). The ML (x-axis) contribution was greater at L4 (24%–28%) than L2 (6%–16%). Percentages are based on mean acceleration magnitudes across all thrusts in each condition. **(A)** L2 Facet. **(B)** L2 Spinous. **(C)** L4 Facet. **(D)** L4 Spinous.

The directional distribution of acceleration at L2 was broadly similar for both contact types. For facet contacts, the axial dominance proportions were: y-axis (PA) = 54%, z-axis (CC) = 30%, x-axis (ML) = 16%. For spinous contacts, the corresponding proportions were: y-axis = 60%, z-axis = 34%, x-axis = 6% (Figure 4). Both contact types thus produced a predominantly posterior-anterior response at L2, with approximately one-third of the total acceleration directed cranial-caudally.

At L4, the directional distributions showed greater divergence between contact types. Facet contacts produced: y-axis = 59%, z-axis = 17%, x-axis = 24%. Spinous contacts produced: y-axis = 67%, z-axis = 5%, x-axis = 28%. The most notable difference was in the z-axis (CC) contribution, which decreased from 19% for facet contacts to 5% for spinous contacts at L4. This pattern is consistent with geometric redistribution of mechanical energy as it propagates through intervertebral structures, though the small sample precludes definitive mechanistic conclusions. Both contact types showed an increased medial-lateral contribution at L4 (26%–28%) compared with L2 (6%–16%), which may reflect the coupled rotational motions that accompany axial transmission through the lumbar facet joints.

### 3.4 Neuromuscular responses (secondary descriptive outcome)

The following EMG observations are presented as secondary descriptive findings. With only one channel (L4 right) available across all three force-usable patients and the remaining three channels limited to two patients, formal inferential comparisons were not possible for most channels. These observations are included for completeness and to inform the design of future studies with dedicated EMG protocols. EMG responses were examined with the caveat that channel availability was unequal across the four recording sites. The L4 right (inferior right) channel had complete data for all 1,549 force-usable thrusts, while the remaining three channels (L2 left, L2 right, L4 left) were available for 728 thrusts (47% of the total), with data limited to Patients 03 and 04. This disparity in sample size limits direct comparison across channels and requires that multi-channel results be interpreted cautiously.

Table 3 presents the EMG P2P amplitudes by contact type for all available channels with the corresponding thrust counts. For the L4 right channel (n = 1,549), facet contacts produced modestly larger P2P EMG amplitudes than spinous contacts, though the patient-level comparison did not reach statistical significance. For the remaining channels (n = 728), the limited number of patients contributing data (n = 2) precluded formal paired testing. These results should be considered preliminary and are reported primarily for completeness.

**TABLE 3:** EMG P2P amplitude (mV) by contact type and channel.

EMG channel	n thrusts	Facet	Patient d	Patient P	Spinous	Patients
L2 left (Sup L)	728	4,971 ± 2,190	-	-	5,674 ± 2,258	P03, P04
L2 right (Sup R)	728	1,733 ± 1,174	-	-	2,426 ± 1,282	P03, P04
L4 left (Inf L)	728	1,118 ± 953	-	-	1,449 ± 1,056	P03, P04
L4 right (Inf R)	1,549	429 ± 374	0.62	0.392	371 ± 373	P02–P04

Values are thrust-level means ± SD. EMG data for channels other than L4 Right were limited to Patients 03 and P04 (partial) due to signal clipping, totaling 728 thrusts. Patient d and P reported only for L4 Right (n = 3 patients); other channels had n = 2 patients (insufficient for paired testing). P value from patient-level paired t-test.

### 3.5 Segmental level comparison: L3 vs. L5 spinous process

**FIGURE 5:**
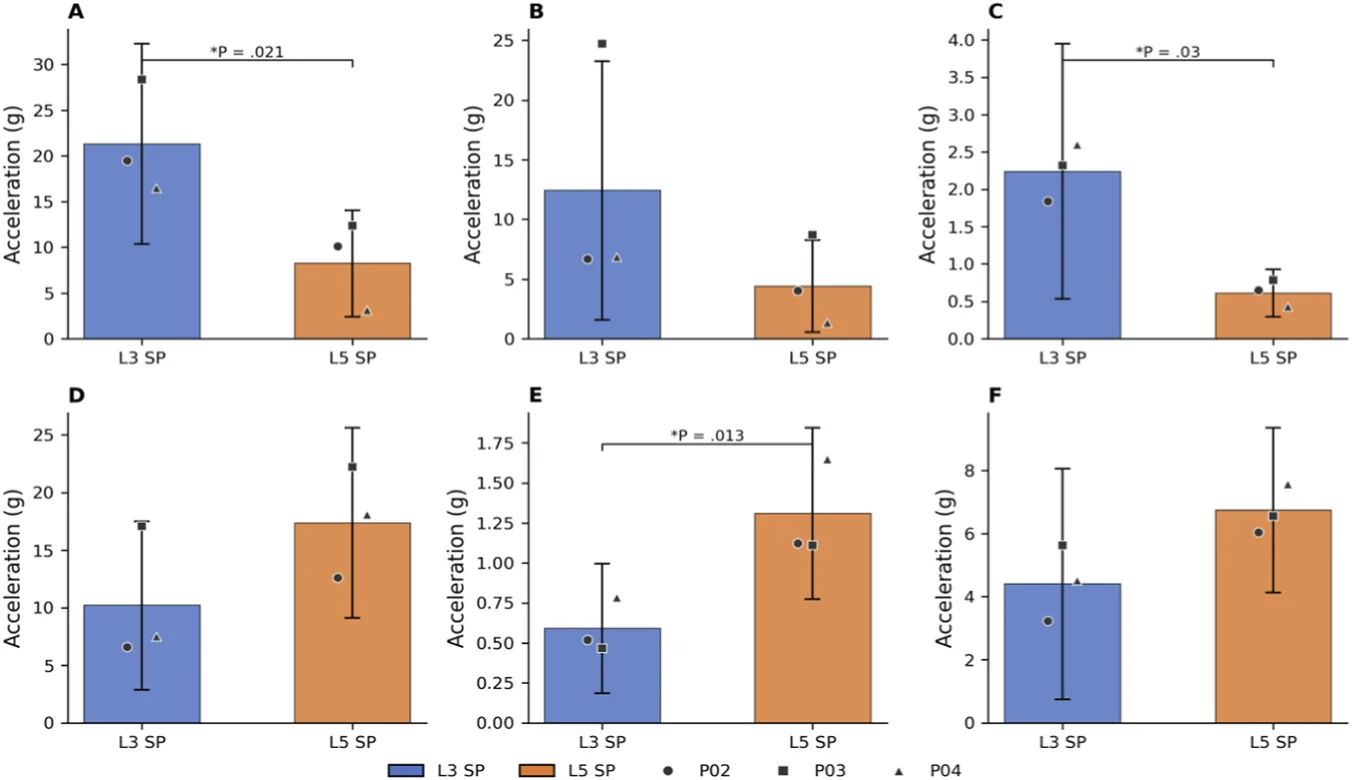
Comparison of L3 vs. L5 spinous process contacts (Patients 02–04, spinous contacts only). L3 contacts (n = 275) produced significantly larger L2 PA acceleration than L5 contacts (n = 260; 21.30 vs. 8.23 g, *P* = 0.021, d = 3.90), consistent with proximity-dependent attenuation. Conversely, L5 contacts produced larger L4 CC acceleration (1.31 vs. 0.59 g, *P* = 0.013, d = −5.04). Asterisks (*) denote patient-level *P* < 0.05. Error bars represent ±1 SD. **(A)** L2 PA. **(B)** L2 CC. **(C)** L2 ML. **(D)** L4 PA. **(E)** L4 CC. **(F)** L4 ML.

The comparison of L3 versus L5 spinous process contacts yielded the largest and most directionally consistent effect sizes in the study, with several comparisons reaching patient-level statistical significance despite the small sample (Table 4; Figure 5). L3 contacts produced substantially greater L2 posterior-anterior acceleration than L5 contacts in all three patients (21.3 vs. 8.2 g, *d* = 3.90, patient-level *P* = 0.021). L3 contacts also produced greater L2 medial-lateral acceleration (2.24 vs. 0.61 g, *d* = 3.27, patient-level *P* = 0.030). Conversely, L5 contacts produced greater L4 cranial-caudal acceleration than L3 contacts (1.31 vs. 0.59 g, *d* = −5.04, patient-level *P* = 0.013).

**TABLE 4:** L3 vs. L5 spinous process comparison (Patients 02–04, spinous contacts only).

Variable	L3 SP	L5 SP	Patient d	Patient P
L2 y-axis, PA (g)	21.30 ± 10.94	8.23 ± 5.82	3.90	0.021
L2 z-axis, CC (g)	12.43 ± 10.83	4.40 ± 3.85	1.14	0.186
L2 x-axis, ML (g)	2.24 ± 1.71	0.61 ± 0.32	3.27	0.030
L4 y-axis, PA (g)	10.21 ± 7.33	17.36 ± 8.25	−2.46	0.051
L4 z-axis, CC (g)	0.59 ± 0.41	1.31 ± 0.54	−5.04	0.013
L4 x-axis, ML (g)	4.40 ± 3.66	6.74 ± 2.61	−1.94	0.078
Transfer ratio (L4z/L2z)	0.094 ± 0.140	0.619 ± 0.503	−0.93	0.250

P values from patient-level paired t-test (n = 3 patients). Effect sizes (Cohen’s d) computed at the patient level.

These results are consistent with the expected proximity-dependent attenuation of mechanical transmission. L3 is situated between the two instrumented vertebrae (L2 and L4), whereas L5 is caudal to both. The inter-vertebral transfer ratio (L4 z/L2 z) was 0.094 for L3 contacts (indicating 90.6% attenuation from L2 to L4) compared with 0.619 for L5 contacts (indicating 38.1% attenuation). These values reflect the geometric reality that L3 contacts are one segment from L2 and three segments from L4, while L5 contacts are three segments from L2 but only one from L4, producing a reversal in the relative magnitudes of acceleration at the two instrumented levels.

### 3.6 Facet bilateral symmetry

**FIGURE 6:**
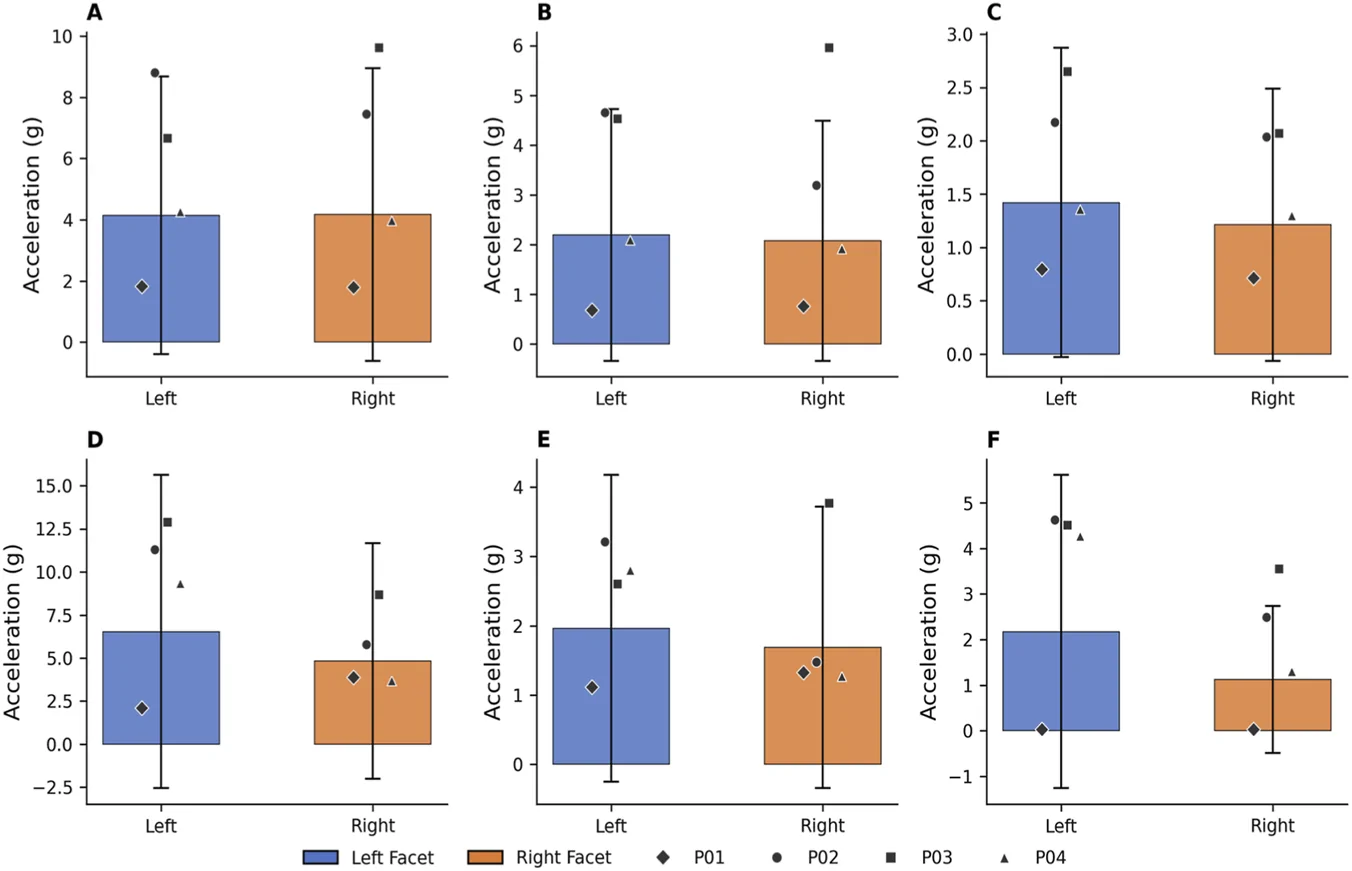
Bilateral facet symmetry: left vs. right facet contacts (all 4 patients, n = 1,090 left, n = 1,042 right). No statistically significant differences were detected between sides at the patient level for any triaxial acceleration channel (all *P* > 0.10). Error bars represent ±1 SD. **(A)** L2 PA. **(B)** L2 CC. **(C)** L2 ML. **(D)** L4 PA. **(E)** L4 CC. **(F)** L4 ML.

No statistically significant bilateral differences were detected between left and right facet contacts at the patient level for any vertebral acceleration axis or EMG channel (Table 5; Figure 6). The small patient sample limits the statistical power to detect subtle bilateral asymmetries, and the absence of significant differences should not be interpreted as evidence of true bilateral equivalence. These results suggest that, at the resolution achievable with 3–4 patients, left and right facet contacts produce broadly comparable mechanical and neuromuscular responses.

**TABLE 5:** Bilateral facet symmetry: left vs. right facet contacts.

Variable	Left facet	Right facet	Patient P
L2 y-axis, PA (g)	4.15 ± 4.53	4.17 ± 4.78	NS
L2 z-axis, CC (g)	2.19 ± 2.53	2.08 ± 2.41	NS
L4 y-axis, PA (g)	6.53 ± 9.09	4.82 ± 6.84	NS
L4 z-axis, CC (g)	1.96 ± 2.21	1.69 ± 2.03	NS
EMG L4 right (mV)	436 ± 264	455 ± 253	NS

NS, not significant at α = 0.05 (patient-level paired t-test). The small sample (n = 3–4 patients) limits power to detect subtle bilateral asymmetries.

## 4 Discussion

This exploratory study characterized the mechanical transmission of impulsive instrument-assisted spinal manipulation in the *in vivo* human lumbar spine using intraosseous accelerometry and needle electromyography. Two principal findings emerged. First, spinous process contacts produced larger posterior-anterior acceleration at the nearest instrumented vertebra (L2), while the L4 response was axis-dependent: facet contacts produced larger cranial-caudal acceleration at L4, but spinous contacts produced larger posteroanterior and medial-lateral acceleration at L4. Second, the most robust finding was the proximity-dependent transmission observed for spinous process contacts at different vertebral levels, with L3 contacts producing significantly greater L2 acceleration than L5 contacts in all patients.

The observation that spinous process contacts produced larger PA acceleration at L2 is consistent with the anatomical rationale for contact site selection. The spinous process is a midline bony prominence connected to adjacent vertebrae through the supraspinous and interspinous ligament complex. Force applied to this structure is channeled primarily in the sagittal plane, producing predominant PA vertebral motion at the nearest level. By contrast, facet contacts apply force to a laterally positioned articular surface situated deep to the paraspinal musculature. The resulting mechanical input may be distributed across a broader range of tissue structures, including the synovial joint capsule, adjacent musculature, and the facet articular surfaces themselves, potentially producing a more distributed spatial response pattern.

These *in vivo* observations are broadly consistent with cadaveric findings from Funabashi and colleagues (Funabashi et al., 2018; Funabashi et al., 2016; Funabashi et al., 2022), who demonstrated that the site of SMT application alters the distribution of internal spinal tissue loading. In their cadaveric model, different contact points produced different patterns of facet joint, intervertebral disc, and ligament strain, supporting the concept that contact site selection modulates the mechanical pathway through which force reaches the spinal structures. Kawchuk and coworkers (Kawchuk et al., 2010) similarly demonstrated using robotic serial dissection that the removal of successive tissue layers progressively altered the force-displacement response during SMT, indicating that multiple tissue elements contribute to the overall mechanical transmission pathway. Our *in vivo* data extend these cadaveric findings by showing that different contact sites produce measurably different acceleration response patterns at instrumented vertebrae in living subjects.

The proximity-dependent attenuation observed for L3 versus L5 spinous contacts represents the largest and most directionally consistent finding in this study. The inter-vertebral transfer ratio reversed from 0.094 (L3 contacts: 90.6% attenuation from L2 to L4) to 0.619 (L5 contacts: 38.1% attenuation), reflecting the geometric reality that each contact level is one segment from one accelerometer and three segments from the other. This distance-dependent attenuation is consistent with animal model findings by Pickar and colleagues (Pickar et al., 2007; Reed and Pickar, 2015), who demonstrated that paraspinal muscle spindle afferent responses during SMT are graded by the distance between the contact site and the recording electrode. Together, these findings from independent experimental paradigms support a general principle that mechanical and neurophysiological responses to SMT attenuate with increasing distance from the point of force application, though the rate and pattern of attenuation likely depend on the anatomical structures traversed.

The stylus accelerance values (3.70 kg^-1^ for facet, 5.36 kg^-1^ for spinous contacts) fall within the range of previously published PA stiffness indices for the lumbar spine. Colloca and Keller (Colloca and Keller, 2001) reported stylus accelerance values of 5.5–7.0 kg^-1^ for lumbar spinous process contacts in intact, non-surgical subjects using the same instrument platform. The somewhat lower values in the present intraoperative sample may reflect patient anatomical or pathological differences that contribute to spinal stiffness, or differences in patient positioning and anesthetic state. Keller and colleagues (Keller et al., 2003a; Keller et al., 2003b) have also documented the relationship between applied force magnitude and vertebral acceleration response, providing additional context for interpreting the driving-point properties reported here.

The question of mechanical specificity in SMT has been a recurring theme in the biomechanics literature (Colloca et al., 2006; Kawchuk and Herzog, 1993; Colloca et al., 2000; Keller et al., 2003a; Keller et al., 2003b). Colloca et al. (2006) and Colloca et al. (2000) showed that vertebral acceleration responses during instrument-assisted SMT vary systematically with both the force amplitude and the vertebral level targeted, suggesting that the mechanical response is not a generic whole-spine phenomenon but rather reflects the local and regional biomechanical properties of the contacted segment. The present findings extend this concept by suggesting that the type of anatomical structure contacted (spinous process vs. facet joint) may also contribute to the specificity of the mechanical response, though replication in larger samples is needed to confirm this observation.

It is important to note that the impulsive forces delivered by the Impulse iQ instrument differ substantially from manual SMT in both temporal profile and force magnitude. Manual high-velocity, low-amplitude thrusts typically have durations of 100–200 milliseconds and peak forces of 200–600 N (Triano et al., 2012; Descarreaux and Dugas, 2010), compared with the approximately 2-millisecond impulse duration and 100–400 N peak forces of the instrument. The extent to which the contact-site-dependent transmission patterns observed here generalize to manual SMT delivery remains an open question, though the underlying anatomical pathways are presumably shared regardless of the temporal characteristics of the applied force.

### 4.1 Limitations

Several important limitations must be considered when interpreting these results. First, the sample of 3–4 patients provides very limited statistical power and precludes generalization to broader clinical populations. In addition, Patient 01 was excluded from all force-dependent analyses due to a force transducer malfunction, reducing the force-usable sample to three patients. This further limits the precision of force-normalized metrics (accelerance, vertebral accelerance) and the associated statistical comparisons. The study is explicitly exploratory, and the observed patterns should be treated as hypotheses to be tested in larger samples rather than established biomechanical principles.

No formal multiple testing correction was applied across the numerous comparisons reported in Tables 1–5. With so few degrees of freedom, standard corrections (e.g., Bonferroni, Holm) would render essentially all comparisons non-significant regardless of the underlying effect magnitude, thereby eliminating the study’s ability to identify potentially meaningful patterns. However, the absence of correction means that some of the nominally significant findings (at α = 0.05) may represent chance observations. The reader should interpret individual *P* values in the context of the full pattern of results, the consistency of effects across patients, and the accompanying effect sizes, rather than treating any single comparison as confirmatory.

Second, data were collected in an intraoperative setting under general anesthesia, which may attenuate reflexive neuromuscular responses and alter spinal mechanical properties compared with awake, non-surgical conditions. These data were collected prior to any surgical exposure, so there is no concern of the surgical procedure influencing force transmission pathways.

EMG data were limited by incomplete channel availability. Only the L4 right channel had data across all force-usable thrusts, with the remaining three channels available for only 47% of thrusts (limited to two patients). This substantially limits the conclusions that can be drawn regarding the neuromuscular component of the transmission response. The EMG findings are presented as descriptive observations rather than tested hypotheses, given the channel availability constraints described in Supplementary Table S1.

It should be noted that the intraoperative bone-pin methodology employed in this study is one of the most technically demanding and ethically constrained protocols in musculoskeletal biomechanics research. The protocol requires identification of patients undergoing elective lumbar surgery who consent to the insertion of additional intraosseous instrumentation (Steinmann pins with triaxial accelerometers and bilateral needle EMG electrodes) under general anesthesia, solely for research purposes. The associated ethical constraints, surgical logistics, patient recruitment challenges, and the progressive discontinuation of bone-pin research worldwide mean that datasets of this kind are exceptionally rare. To our knowledge, the present study and our previous work (Colloca et al., 2000; Keller et al., 2003a; Keller et al., 2003b) represent the only *in vivo* human bone-pin accelerometry studies of instrument-assisted spinal manipulation conducted to date. Similar pioneering bone-pin studies in other domains of spine biomechanics (Rozumalski et al., 2008; MacWilliams et al., 2014; Schmid et al., 2024) have also employed small samples (n = 5–10) and have been widely cited as foundational contributions despite their sample sizes, because they provide measurements that are impossible to obtain by any other means. The present findings should therefore be interpreted not as unreliable due to small sample size, but rather as the first direct *in vivo* measurements of their kind, reported with full transparency about the achievable statistical resolution, and intended to generate hypotheses for testing in complementary non-invasive experimental designs.

It should be noted that the L3 versus L5 segmental comparison confounds vertebral level with accelerometer proximity. L3 is situated between the instrumented vertebrae (L2 and L4), while L5 is caudal to both. The observed differences in transmission therefore reflect both the biomechanical properties specific to each vertebral level and the geometric relationship between the contact site and the measurement locations. The present data cannot distinguish between these explanations. However, it should be noted that the number of intervening motion segments is itself a biomechanical property of the transmission pathway, as each intervertebral disc-facet complex contributes frequency-dependent attenuation; the observed distance-attenuation relationship is therefore inherent to spinal anatomy rather than a design limitation. Future studies employing multi-level accelerometry arrays or finite element modeling would be needed to isolate the contribution of inter-segmental distance from level-specific tissue properties. Last, although conditions were delivered in randomized trial order with recovery intervals between trials, progressive tissue changes during the experimental session (e.g., viscoelastic creep, tissue hydration changes, or residual neuromuscular effects from preceding thrusts) cannot be excluded. Additionally, with only 3–4 patients, individual differences in anatomy, pathology, and tissue properties cannot be systematically assessed. Replication in a larger cohort is needed to confirm the observed patterns and to evaluate potential moderating factors.

### 4.2 Clinical implications

This study did not evaluate clinical outcomes, and direct translation of these intraoperative biomechanical findings to clinical technique selection requires validation in awake, symptomatic patient populations. Nevertheless, the biomechanical observations may inform the development of mechanistic hypotheses. At the level of biomechanical observation, the data suggest that contact site selection influences the directional character and spatial distribution of the vertebral mechanical response. At the level of mechanistic hypothesis, if different contact sites produce different mechanical transmission patterns (as these data suggest), then they may also produce different neurophysiological and clinical effects, though this hypothesis remains untested. At the level of clinical speculation, these findings raise the possibility that contact site selection could be optimized based on the type and location of the biomechanical dysfunction being addressed, but this speculation requires substantial additional evidence before clinical adoption.

Future studies should examine whether the contact-site-dependent transmission patterns observed here translate to measurable differences in clinical outcomes. Such studies would require larger samples, non-surgical settings, validated outcome measures, and randomized designs that isolate the effect of contact site from other treatment parameters.

## 5 Conclusion

In this exploratory *in vivo* study of four patients undergoing lumbar surgery, two patterns of mechanical transmission were observed that differed by contact site. Spinous process contacts produced larger posterior-anterior acceleration at the nearest instrumented vertebra (L2), while facet contacts produced relatively larger cranial-caudal responses at the more distal vertebra (L4). Proximity-dependent inter-vertebral transmission was observed for spinous contacts, with L3 producing significantly greater mechanical response at L2 than L5 (patient-level *P* = 0.021). No significant bilateral asymmetry was detected for facet contacts at the patient level. Stylus accelerance values (3.70–5.36 kg^-1^) were consistent with published normative values, supporting the validity of the corrected signal processing pipeline. These findings suggest that contact site selection influences the mechanical transmission pathway during impulsive spinal manipulative therapy and may help generate hypotheses for future clinical studies examining the relationship between contact site selection and therapeutic outcomes. Replication in larger samples is needed before clinical translation.

## Data Availability

The raw data supporting the conclusions of this article will be made available by the authors, without undue reservation.
