# Supplementary material for: Mechanical transmission of impulsive instrument-assisted spinal manipulation: contact-site-dependent biomechanical coupling in the in vivo human lumbar spine
Source: Front Bioeng Biotechnol. 2026 Aug 13;14:1849593. doi: 10.3389/fbioe.2026.1849593 (PMC13519242; doi:10.3389/fbioe.2026.1849593)
Supplement: Supplementary file 1 [file Table1.docx]

**Table S1.** Data availability matrix by measurement channel and patient. Values indicate the number of thrusts with usable data for each channel (percentage of patient total). Shaded cells indicate channels affected by data quality exclusions.

| **Measurement Channel** | **Patient 01 (n = 1,685)** | **Patient 02 (n = 530)** | **Patient 03 (n = 498)** | **Patient 04 (n = 521)** | **Exclusion Reason** |
| --- | --- | --- | --- | --- | --- |
| **Force Transducer** | | | | | |
| Applied force (N) | 0/1,685 (0%) | 530/530 (100%) | 498/498 (100%) | 521/521 (100%) | P01: instrument malfunction (~5–9 N across all settings) |
| **Vertebral Accelerometers (L2, triaxial)** | | | | | |
| L2 posteroanterior (PA) | 1,685 (100%) | 530 (100%) | 498 (100%) | 521 (100%) | — |
| L2 cranial-caudal (CC) | 1,685 (100%) | 530 (100%) | 498 (100%) | 521 (100%) | — |
| L2 medial-lateral (ML) | 1,685 (100%) | 530 (100%) | 498 (100%) | 521 (100%) | — |
| **Vertebral Accelerometers (L4, triaxial)** | | | | | |
| L4 posteroanterior (PA) | 1,685 (100%) | 530 (100%) | 498 (100%) | 521 (100%) | — |
| L4 cranial-caudal (CC) | 1,685 (100%) | 530 (100%) | 498 (100%) | 521 (100%) | — |
| L4 medial-lateral (ML) | 1,685 (100%) | 530 (100%) | 498 (100%) | 521 (100%) | — |
| **Paraspinal EMG (bilateral needle electrodes)** | | | | | |
| L2 left | —ᵃ | 0/530 (0%) | 498/498 (100%) | 230/521 (44%) | P02: signal clipping; P04: partial clipping |
| L2 right | —ᵃ | 0/530 (0%) | 498/498 (100%) | 230/521 (44%) | P01: no data; P02: signal clipping; P04: partial |
| L4 left | —ᵃ | 0/530 (0%) | 498/498 (100%) | 230/521 (44%) | P01: no data; P02: signal clipping; P04: partial |
| L4 right | 1,685 (100%) | 530/530 (100%) | 498/498 (100%) | 521/521 (100%) | — |

^ᵃ^ Patient 01 EMG data were available for L2 left (457/1,685 thrusts) and L4 right (1,685/1,685 thrusts) but are excluded from force-dependent analyses due to force transducer malfunction.

**Summary of data exclusions and analytic impact:**

**1. Force transducer malfunction (Patient 01):** Patient 01’s force transducer produced anomalous readings (~5–9 N across all force settings, compared with expected values of 100–400 N). Patient 01 was excluded from all force-dependent analyses (accelerance, vertebral accelerance, force comparisons), reducing the force-usable sample from 3,234 to 1,549 thrusts (Patients 02–04). Patient 01 data were retained for acceleration-only analyses.

**2. EMG signal clipping (Patients 01, 02, 04):** Three of four EMG channels (L2 left, L2 right, L4 left) were affected by signal clipping in Patients 02 and partially in Patient 04. Only the L4 right channel provided complete data across all 1,549 force-usable thrusts (100%). The remaining three channels were available for 728/1,549 thrusts (47%), limited to Patients 03 and 04. EMG analyses using the three affected channels are therefore based on a reduced sample.

**3. Accelerometer saturation:** All six vertebral accelerometer channels provided complete data for all thrusts. However, the triaxial accelerometers (Crossbow CXL10HF3, nominal range ±10 g) occasionally produced responses exceeding the nominal measurement range. For L2, 14.2% of thrusts exceeded 10 g peak-to-peak on at least one axis; for L4, fewer than 2% approached the ceiling. A sensitivity analysis excluding these ceiling-adjacent thrusts did not change the direction or significance of any patient-level comparison.
